# Epigenomic annotation of noncoding mutations identifies mutated pathways in primary liver cancer

**DOI:** 10.1371/journal.pone.0174032

**Published:** 2017-03-23

**Authors:** Rebecca F. Lowdon, Ting Wang

**Affiliations:** Center for Genome Sciences and Systems Biology, Department of Genetics, Washington University in St. Louis, Saint Louis, Missouri, United States of America; Pohang University of Science and Technology, REPUBLIC OF KOREA

## Abstract

Evidence that noncoding mutation can result in cancer driver events is mounting. However, it is more difficult to assign molecular biological consequences to noncoding mutations than to coding mutations, and a typical cancer genome contains many more noncoding mutations than protein-coding mutations. Accordingly, parsing functional noncoding mutation signal from noise remains an important challenge. Here we use an empirical approach to identify putatively functional noncoding somatic single nucleotide variants (SNVs) from liver cancer genomes. Annotation of candidate variants by publicly available epigenome datasets finds that 40.5% of SNVs fall in regulatory elements. When assigned to specific regulatory elements, we find that the distribution of regulatory element mutation mirrors that of nonsynonymous coding mutation, where few regulatory elements are recurrently mutated in a patient population but many are singly mutated. We find potential gain-of-binding site events among candidate SNVs, suggesting a mechanism of action for these variants. When aggregating noncoding somatic mutation in promoters, we find that genes in the ERBB signaling and MAPK signaling pathways are significantly enriched for promoter mutations. Altogether, our results suggest that functional somatic SNVs in cancer are sporadic, but occasionally occur in regulatory elements and may affect phenotype by creating binding sites for transcriptional regulators. Accordingly, we propose that noncoding mutation should be formally accounted for when determining gene- and pathway-mutation burden in cancer.

## Introduction

Cancer genomics suffers from a dramatic signal to noise problem, where the majority of somatic mutations are not expected to cause cancer phenotypes, but to be passenger mutations that do not contribute to selective growth advantage [1–3]. The challenge of identifying mutations that change cancer phenotype is especially difficult in the noncoding genome: whereas over 50 years of molecular genetics research has given cancer investigators a toolkit for understanding the deleteriousness of coding mutation, the same code book does not exist for noncoding mutations. Instead, anecdotal instances of oncogenic noncoding mutations in the cancer literature include a variety of mechanisms, including transcription factor binding site creation (or deletion) by point mutation [4–8], modulation of splicing events [9], enhancer hijacking by structural rearrangements [10,11], or abrogation of chromatin neighborhoods by disruption of cohesion binding sites [12]. Considering the mechanistic diversity of noncoding mutation, we interrogated a single route of oncogenic gene regulation: appropriation of regulatory elements from heterologous cell types. Anecdotal examples of such events have been characterized previously [10,13]. In addition, a recent comprehensive analysis of regulatory mutation across cancer types suggested that noncoding mutation be more consequential in the context of cancer than previously understood [14]. Therefore we aimed to increase our sensitivity for recovering regulatory element hijacking events by functional noncoding mutations by focusing our analyses on point mutations that occur in epigenetically-defined regulatory elements.

As the importance of regulatory variation has become illuminated [15,16] several tools for detecting deleterious noncoding mutation have been developed in recent years. These tools implement empirical scoring algorithms and machine learning approaches to determining functional noncoding variants. These algorithms use a combination of negative selection [17,18], mutation recurrence [17], and/or functional element annotation data [18–21] (e.g. from the ENCODE Project [15]) to predict noncoding variant significance [22]. In the study presented here, we expand noncoding variant annotation to include the wealth of epigenomic data, now publically available by resources such as the Roadmap Epigenomics Project [16,23]. Epigenome annotation data allow us to investigate the hypothesis that somatic mutations might activate transcriptional regulatory programs not native to the tumor cell type of origin.

**Fig 1:**
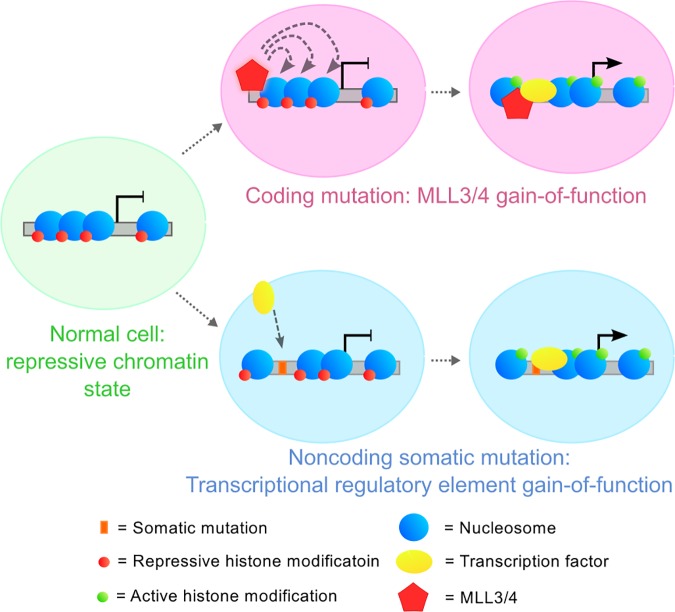
Models for regulatory element involvement in cancer. In the *trans*-model of cancer enhancers, somatic mutation to a chromatin modifier gene, here *MLL3/4* (red pentagon), results in that chromatin modifier binding more tightly to a DNA-bound transcription factor (yellow oval) and aberrantly creates a persistently open chromatin state, up-regulating the target gene. In the *cis*-model of cancer enhancers, a somatic mutation to a noncoding regulatory element (orange bar) creates the same open chromatin state, perhaps by creating a binding site for a transcription factor that is recruited to the locus and facilitates opening local chromatin.

One model of regulatory element-mediated oncogenesis in the literature is the cancer enhancer model (**Fig 1**). In the cancer enhancer model, coding mutations can have oncogenic effects by mis-regulation of the epigenome. For example, mutation of chromatin modifier genes (for example, *mixed lineage leukemia* (*MLL*) family genes) may adjust the affinity of transcriptional activators for cognate enhancers, driving over-expression of a proto-oncogene [24]. Alternately, in a tumor suppressor context, mutated chromatin modifiers may reduce affinity of trans-activators for enhancers, in either case leading to tumor progression [24].

Analogously, we propose the *cis*-cancer enhancer model, whereby somatic mutation of regulatory elements changes their regulatory potential (**Fig 1**). The *cis*-cancer enhancer model predicts that functional noncoding mutation may activate transcriptional regulatory programs intrinsic to heterologous cells. In our model, noncoding somatic mutation might change the regulatory potential of an element by creating a binding site for a DNA-binding protein, subsequently allowing the protein to bind DNA and recruit other chromatin modifiers. Such activity is reminiscent of pioneer factor action, which subsequently recruits transcriptional activators, as has been demonstrated to occur in the context of breast cancer mutations that create FOXA1 binding sites [4]. By modulating the epigenetic status of the regulatory DNA fragment, somatic mutation “hijacks” regulatory element activity intrinsic to another cell type.

Accordingly, here we use epigenomic annotation from diverse cells and tissues to test the hypothesis that noncoding mutation activates regulatory elements used in heterologous cells. We found that after filtering, approximately 40.5% of noncoding variants fall in transcriptional regulatory elements. Subsequently, we found widespread potential gain or loss of transcription factor binding sites, suggesting specific mechanisms by which noncoding mutation may influence cancer phenotype and progression. Last, we determined that noncoding regulatory mutations in primary liver cancer (PLC) occur in promoters for genes involved in transcriptional misregulation in cancer, ERBB signaling, and MAPK signaling pathways.

Genome-wide studies of regulatory mutation in cancer have analyzed noncoding mutation from a pan-cancer perspective [25–27]. These studies have found repeatedly a limited set of candidate noncoding variants that are responsible for phenotype in the pan-cancer context. Fewer have queried the effect of noncoding mutation in cancer on a single disease basis [28–34]. In the present study, we aimed to increase our specificity by focusing on a single disease, we chose to study PLC for two reasons: first, normal liver tissue is relatively homogeneous, making determination of regulatory elements easier. Second, there are many publically available liver cancer samples, and a large sample size is necessary in order to detect rare events.

## Results

### Isolating putatively functional noncoding single nucleotide variants

The Catalog of Somatic Mutations in Cancer (COSMIC) project houses publically available cancer genetics data [35]. The repository includes data from a variety of diseases and various assay types (e.g. whole genome resequencing, ExomeSeq). For the present work, we used the noncoding variants dataset from the COSMIC Genomes project.

**Fig 2:**
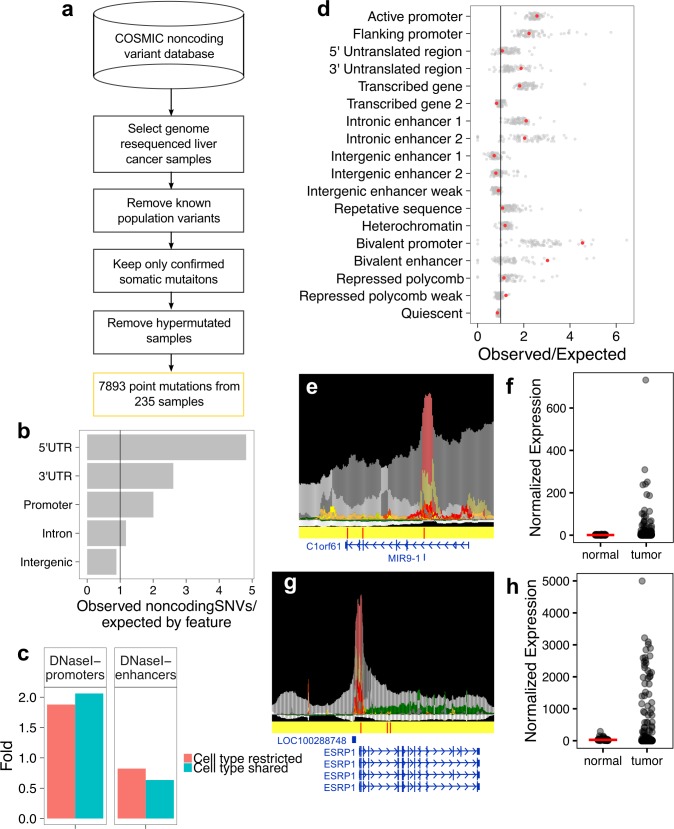
PLC NCVs occur more often than expected in heterologous cell type-specific regulatory elements. (a) Filtering strategy for SNVs from whole genome resequenced samples in COSMIC. (b) Annotation of filtered SNVs by UCSC known genes. (c) SNVs in cell type restricted or shared DNaseI promoters or enhancers. Y-axis is fold observed over expected, based on background distribution of cell type restricted or shared DHSs. (d) Observed versus expected SNVs in each ChromHMM-18 state in each of the 78 Roadmap cells and tissues with available data. Orange dot is primary liver sample (Roadmap E066); gray dots are the other 77 Roadmap samples; black line is 1. (e) Browser view of *C1orf61* locus and three regulatory elements mutated in three unique samples. The top track is the Epilogos track (http://compbio.mit.edu/epilogos/), which provides a visualization of the chromatin state models for several cell types at once. The presented track depicts the ChromHMM-18 state model 127 Roadmap cell types (primary and cell lines) at a 200bp resolution. Red and orange colors represent active promoter annotations; light green and yellow colors represent genic enhancers and enhancers, respectively; pink and beige are bivalent states; grays are repressed Polycomb states. Middle track: Positions of PLC WGS SNVs (red lines) on a yellow background. Bottom track: RefSeq genes track. (f) Expression from TCGA PLC tumor and matched normal samples for *C1orf61*. Red line = median expression for normal samples. (g) Browser view for *ESRP1* and three regulatory elements mutated in three unique samples. Tracks are as in (e). (h) Expression as in (f) for *ESRP1*.

To isolate putatively functional noncoding single nucleotide variants (SNVs) in the COSMIC dataset, we took a stringent filtering approach (**Fig 2A; Methods**). After isolating noncoding SNVs from primary liver cancer (PLC) samples, we removed variants at positions of known population variants and kept only variants that were confirmed somatic (e.g. not observed in the matched normal genome) and that were discovered from whole genome resequencing (WGS) (**Methods**). We focused our analysis on WGS-derived variants because we wanted an unbiased interrogation of somatically mutated genome-wide regulatory elements. Because the WGS data were collected over a long period of time and across different research projects, information about sequencing coverage and depth were not directly available from COSMIC database and presumably variable. Thus, these data do not allow us to assess mutation rate and to derive a complete catalog of non-coding mutations in liver cancer. However, the wide range of samples and high quality of the mutation information in the COSMIC database ensure that our analysis of non-coding mutations in liver cancer is representative.

Next, we determined the distribution of noncoding SNVs per sample ID in COSMIC. Hypermutator phenotypes occur when DNA repair genes have been inactivated and DNA mutation occurs unchecked [36]. To remove noise due to hypermutation, variants from samples with the top 2.5% of SNVs/sample were removed (7 samples with 79817 total SNVs; **S1A Fig**; **Methods**). This noncoding SNV filtering strategy resulted in 7893 noncoding SNVs from 235 unique liver cancer samples in the COSMIC database.

The same strategy applied to ExomeSeq noncoding variants returned 1477249 noncoding SNVs from 789 unique liver cancer samples (**S1B and S1C Fig**). The higher ratio of mutations reported by ExomeSeq presumably reflects much deeper sequencing depth in typical ExomeSeq experiments than in WGS experiments.

All analyses were run on filtered WGS and ExomeSeq SNV sets separately; however all results are for WGS-SNVs unless otherwise noted.

### Genome feature annotation of noncoding single nucleotide variants in liver cancer

Annotating noncoding SNVs by the UCSC known genes annotation set revealed that noncoding somatic mutations were markedly enriched in UTRs and promoters (**Fig 2B**). Promoters and UTRs are sites with a high density of regulatory elements. Thus, noncoding SNVs that passed our filtering strategy were likely enriched in genome regions that host regulatory features.

### Epigenomic annotation of noncoding single nucleotide variants in liver cancer

The Roadmap Epigenome Project generated reference epigenomic datasets for 111 primary human cell types and tissues [16]. Among the data generated were chromatin immunoprecipitation-sequencing (ChIP-seq) for various histone modifications. Histone ChIP-seq data for each tissue were then synthesized by the ChromHMM algorithm to produce a genome-wide annotation of epigenomic status [16]. Other experiments included DNaseI-hypersensitivity sequencing and were conducted on a subset of tissues.

DNaseI hypersensitive regions are enriched for transcriptional regulatory elements such as enhancers and promoters [37]. To validate that noncoding SNVs delivered by our algorithm were likely to be regulatory, we analyzed the SNV locations in the context of the Roadmap DNaseI hypersensitivity site (DHS) data. The catalog of DHS regions was collected from the 39 Roadmap Epigenomes for which data were available, and the ChromHMM promoter or enhancer status of these DHS positions was queried in all 111 Roadmap Epigenome primary cell types. Notably, the single primary liver sample in the Roadmap Project did not have DNaseI hypersensitivity in the pan-Roadmap DHS site catalog. However, we wanted to determine if non-liver regulatory element accumulated PLC noncoding mutations. Therefore, we partitioned the DHSs into cell type-shared or cell type-restricted regions, as determined by the Roadmap Project analysis of DHS data (**Methods**). Then we assigned each SNV location to a DHS if it fell in a DHS peak as called by the Roadmap Project (**Methods**).

Noncoding somatic PLC SNVs that passed filtering were found in DHSs were annotated as promoters more often than random expectation (**Fig 2C**). Both cell type-shared and cell type-restricted DNaseI-promoters were somatically mutated more than expected (2.06- and 1.88-fold over expectation based on background, respectively). The enrichment for SNVs in cell type-restricted DNaseI-promoters indicates that promoters not specific to liver sustain regulatory mutations in PLC. Enrichment of cell type-shared promoters reflects mutation of promoters for genes that are constitutively expressed. On the other hand, both cell type shared and cell type restricted DNaseI-enhancers were slightly depleted for somatic mutations (0.62-fold and 0.84-fold compared to background expectation respectively). It is likely that the low fold enrichment for DNaseI-enhancers was due to the large expected value, as DNaseI-annotated enhancers accounted for a large percentage of genome base pairs.

### Primary liver cancer single nucleotide variants are enriched in bivalent chromatin features

We suspected analyzing enhancer chromatin states in finer detail would provide a more nuanced picture of the patterns of somatic regulatory mutation. Thus, we analyzed the filtered noncoding PLC SNVs in the context of the ChromHMM-18 state model for Roadmap Epigenome Project primary tissues. We tabulated the occurrence of liver cancer SNVs in each ChromHMM-18 state in each of the 78 cells and tissues for which data were available and compared this value to the expected number of SNVs, assuming a random mutation distribution (**Fig 2D**; **Methods**). Strikingly, we found elevated observed/expected values across most tissues analyzed in regulatory ChromHMM states, including active promoters (1_TssA), flanking promoter regions (2_TssFlnk, 3_TssFlnkU, 4_TssFlnkD), genic enhancers (7_EnhG1, 8_EnhG2), and bivalent states (14_TssBiv, 15_EnhBiv), which have regulatory potential (data in **S3 Table**). Surprisingly, active enhancer states (9_EnhA1, 10_EnhA2) did not have elevated observed/expected values across most cell types. Again, this was likely because these enhancer states occupied a large fraction of the genome (34% of merged epigenome base pairs were annotated as potential enhancer state (active, weak, genic, and bivalent enhancer states) versus 8.4% annotated as potential promoter (active, flanking, and bivalent) (**Methods**).

Specifically in liver sample annotations, we found elevated observed/expected values in active and flanking promoters states (2.56, 2.21 fold enrichment respectively), genic enhancers (2.09, 2.02), and bivalent states (bivalent TSS 4.53, bivalent enhancers, 3.01). The strongest enrichment was for the bivalent transcription start site (TSS) and bivalent enhancer states. Bivalent chromatin is best understood in the embryonic stem cell context, where simultaneous modification of nucleosomes by activating (H3K4me3) and Polycomb-repressive (H3K27me3) histone modifications is thought to keep promoters in a “poised” state until the cell further differentiates [38]. The function of bivalent domains in differentiated cells is less understood, but may enable the cell to respond quickly to environmental stimuli [39,40].

Finding elevated SNVs at bivalent enhancers and promoters prompts the hypothesis that these liable regulatory sites may be central to transcriptional mis-regulation in PLC. For example, dysregulation of bivalent promoters has been shown to lead to oncogene activation in colorectal tumors [41]. Indeed, dysregulation of bivalent domains is a reported phenomenon in cancer genomes [42]. In a process called “epigenome switching,” the Polycomb-deposited repressive histone modification (histone 4 lysine 27 trimethylation) is aberrantly replaced by DNA methylation, which is relatively more stable [43]. It would be interesting to explore if the accumulation of SNVs in bivalent domains is mechanistically linked to recruitment of DNA methyltransferases to these regions in cancer.

Altogether, we find that 40.5% (3200/7893) of SNVs were found in regulatory elements from 78 cell types and tissues genome-wide. Thus, analysis of candidate somatic noncoding mutations in epigenetically defined regulatory elements supports our hypothesis that noncoding somatic mutation may influence cancer phenotype by modulating regulatory elements.

### Patterns of noncoding somatic mutation in regulatory elements mirrors that of coding mutations in genes

Coding mutations in cancer display a stereotypic distribution across genes, where a few genes are recurrently mutated across patients, while a long tail of genes is rarely mutated [2]. This is true for most cancer types, even though the identity of the highly or lowly-mutated genes varies depending on the disease [25,44]. We hypothesized that the distribution of putatively functional regulatory element mutations might mirror the pattern of coding mutation. Indeed, plotting the number of candidate somatic mutations from the COSMIC PLC samples for each regulatory element mapped revealed a striking distribution: one regulatory element is mutated in 16 patients (*p-value* = 2.89713e-14), two regulatory elements are mutated in 7 patients each (*p-value* = 9.952759e-05), and a long tail of individual elements are mutated in 1, 2, or 3 patients (**Table 1**). The most-mutated regulatory element is the *TERT* promoter, which was mutated 16 times at the same position in the ETS binding site, as has been previously reported in the literature [45].

**Table 1:** Number of SNVs per regulatory element.

Number SNVs per element	Number of regulatory elements
1	3035
2	43
3	6
4	2
5	1
7	2
16	1

We sought to connect the candidate noncoding liver mutations to putative target genes. First we assigned the candidate SNVs to regulatory elements, epigenetically defined by the Roadmap Project (**Methods**). Next we assigned each SNV-containing regulatory element to putative target gene promoters (using a +/-35kb window [46]; **Methods**). Based on these target gene assignments, we asked if some target genes have an elevated rate of mutated regulatory elements. We queried the collection of target gene regulatory elements—their promoters and putative distal enhancers—and tabulated the number of somatically mutated regulatory elements associated with each gene (**Table 2**). The distribution is qualitatively similar to that of coding mutations in cancer [47], where in a patient population, a few genes have several noncoding somatic mutations in their regulatory elements, while a long tail of genes have only one mutated regulatory element. We found the distribution of noncoding mutations in regulatory elements follows a similar pattern (**S2 Fig**).

**Table 2:** Number of genes with SNV-containing putative regulatory elements.

Number SNV-containing regulatory element per gene	Number of genes
1	1031
2	52
3	3
5	1

Three genes had three putative regulatory elements with noncoding somatic mutations. One of these was *C1orf61* (**Fig 2E**), which has been characterized as a tumor activator in hepatocellular carcinoma [48]. *C1orf61* is located on 1q22, which experiences copy number amplifications in several cancers including hepatocellular carcinoma [48]. Investigation of the effect of upregulation of *C1orf61* revealed that it was correlated with liver disease and HCC progression, and ectopic expression of C1ORF61 promoted cell proliferation, metastasis, and EMT [48].

In our analysis, each of the three somatically mutated *C1orf61* regulatory elements was found in three unique samples. Importantly, these samples were not recorded with 1q22 amplifications in the COSMIC database, indicating that noncoding regulatory mutation may upregulate *C1orf61* in hepatocellular carcinoma in a similar tumorigenic manner as copy number amplification. We examined The Cancer Genome Atlas expression data for PLC samples and matched normal tissue [27] and found that *C1orf61* expression was elevated in a subset of tumors (**Fig 2F**).

*Epithelial splicing regulatory protein 1* (*ESRP1*) also had three SNV-containing putative regulatory elements (**Fig 2G**). *ESRP1* can promote the epithelial-to-mesenchymal transition (EMT) by regulating alternative splicing of *CD44* [49]. Knockdown of *ESRP1* activity in breast cancer cells restored the non-EMT-inducing isoform of *CD44* and suppressed metastasis [50], evidence that *ESRP1* acts as an oncogene. *ESRP1* acts as a master regulator of EMT in melanoma [51] and somatotroph adenomas [52]. However, upregulation of *ESRP1* is correlated with fewer metastasis and better prognosis in pancreatic ductal adenocarcinoma [53], and acts a tumor suppressor in colorectal cancer [54], reflecting the cell type-specific nature of cancer genes [44].

The filtered PLC SNVs contained three mutations in regulatory elements whose putative target was *ESRP1*. TCGA expression data from PLC and matched normal samples showed that 27% of tumors had elevated expression of *ESRP1* (**Fig 2H**).

The gene with the most somatically mutated regulatory elements was *MAP2K1*, part of the mitogen-activated signaling pathway, which is a central regulator of cell growth. The five *MAP2K1* regulatory elements found mutated in our data set contained seven unique mutated positions in seven samples. At time of writing, *MAP2K1* has not been directly implicated in liver cancer; however the MAPK signaling pathway has been identified as important for PLC [55,56]. *MAP2K1* has been identified as an occasional driver in non-small cell lung cancer [57], and sustained gain-of-function mutations in melanoma [58]. Variation among genes in the MAPK pathway predisposes to colon and rectal cancer, including susceptibility variants in *MAP2K1* [59].

### Regulatory element-annotated single nucleotide variants cause gain-of-binding site events upstream of known oncogenes

Since our hypothesis was that noncoding somatic mutations might activate transcriptional regulatory programs from heterologous cell types, we predicted that functional noncoding mutations in regulatory elements should result in gain-of-function genetic events [60]. Such events may be evident as gain-of-binding site motifs for transcriptional trans-activators.

**Fig 3:**
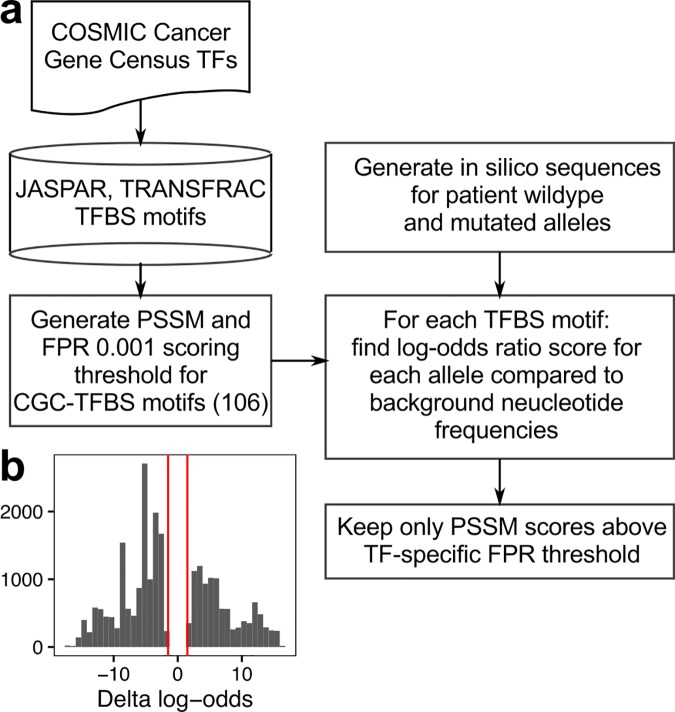
Systematic motif detection identifies oncogenic TFBS gain-of-binding events. (a) Analysis pipeline for detecting motifs from wildtype and mutant allele sequences. (b) Histogram of delta values for WGS SNV allele pairs after filtering to keep only allele pairs with at least one motif score of absolute value ≥ 2.

To test this prediction, we conducted a systematic analysis of somatic SNVs in regulatory elements to look for gain-of-binding site events. First, we queried the COSMIC Cancer Gene Census for transcription factors (termed CGC-TFs), of which there were 93. For these factors, we searched the JASPAR and TRANSFAC motif databases for motifs that are bound by the cognate CGC-TFs; 106 motif position weight matrices (PWMs) were found, including motifs for heterodimers. Finally, for each of the 106 motif PWMs we constructed a position-specific scoring matrix (PSSM) and determined the threshold PSSM value for a false-positive rate of 0.001 (**Fig 3A; Methods**).

We then analyzed each SNV from the filtered COSMIC noncoding variant set that occurred in a regulatory element for its ability to modulate the motif PSSM score. For each SNV, we generated *in silico* wildtype and mutant alleles, using hg19 as the reference (wildtype) allele. Each pair of alleles was scored against each CGC-TF PSSM to obtain a log-odds ratio score compared to a background of genomic nucleotide frequencies (where A = T = 0.3 and G = C = 0.2); only scores passing the CGC-TF-specific threshold were retained.

We determined the delta value for each pair of PSSM scores by subtracting the mutant allele score from the wildtype score (**S3A and S3B Fig**). To enrich our dataset for events with high effect size, we kept only pairs of CGC-TF motif scores where at least one score (wildtype or mutant) was log odds score over background ≥ 2. The resulting distribution reveals that 1234 pairs of wildtype-mutant alleles from whole genome-resequenced samples create potential gain-of-binding site events, in which the mutant allele score is higher than the wildtype allele score for a particular CGC-TF (**Fig 3B**; **S3C Fig**). 1393 allele pairs represent potential loss-of-binding events, where the wildtype allele score was greater than the mutant allele score. Allele pairs residing in promoter regions from ExomeSeq samples resulted in 25600 and 29410 gain and loss of binding sites, respectively. Thus we find a substantial number of potential gain-of-binding site events from candidate noncoding somatic mutations.

**Fig 4:**
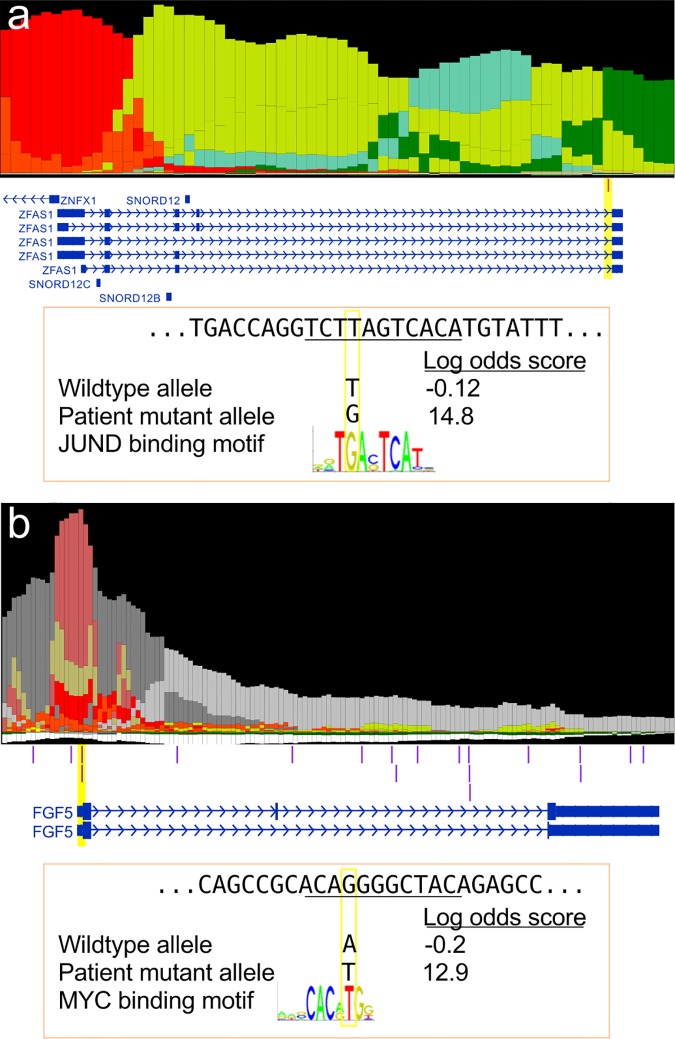
Gain-of-binding site events at known oncogenes. (a) *ZFAS1* locus. SNV occurs in the last intron creating a JUND binding site. (b) *FGF5* locus. SNV in the promoter creates a MYC binding site.

We examined the gain-of-binding site candidates for evidence of oncogenic events. The mutation event with the highest effect size in our dataset was a noncoding mutation in the last intron of *ZFAS1* lncRNA (**Fig 4A**). The *ZFAS1* mutated position is annotated as a genic enhancer in human Mammary epithelial cells (vHMEC) cells by ChromHMM. This T>G mutation creates a strong JUND binding site where the reference sequence is less likely than background to bind JUND (wildtype allele = -0.12; mutant allele = 14.75). Importantly, *ZFAS1* is known to promote metastasis in hepatocellular carcinoma [61,62]. *ZFAS1* is a regulator of normal mammary gland development, where it inhibits miR-150, which in turn inhibits *ZEB1* [61], a regulator EMT [63]. When *ZFAS1* is upregulated in HCC, is hypothesized to act as a sponge to decrease the concentration of miR-150, thereby upregulating *ZEB1*, which induces tumor cell invasion and metastasis in *in vitro* and animal models [62].

Since many SNVs from whole genome-resequenced PLC samples did fall in promoter regions, and promoters are often captured in ExomeSeq data, we expanded the motif mutation analysis to promoter ExomeSeq variants from COSMIC PLC samples. Among the ExomeSeq SNVs, we find a COSMIC patient sample with an A>T mutation in the *FGF5* promoter that creates a MYC binding site (**Fig 4B**). The somatic mutation creates a binding site where the reference sequence is slightly less likely than background to bind MYC (wildtype allele = -0.2; mutant allele = 12.9). *FGF5* is a known oncogene in glioblastoma where it promotes proliferation and inhibits apoptosis [64].

Thus, at least two known oncogenes were recovered in our gain-of-binding candidate somatic mutations. These events suggest that noncoding mutation may mimic oncogenic coding mutations by upregulating proto-oncogenes. Importantly, such gain-of-function mutations may occur at regulatory elements not annotated in the cancer tissue-of-origin (in this case liver) but in regulatory elements active in other cell types (for example, *ZFAS1* in breast tissue).

### Noncoding mutations add to pathway level mutation burden

An important aspect of cancer genomics is that deleterious mutations can inactivate a pathway at several points [44]. For example, in colorectal cancer, *BRAF* mutations are mutually exclusive with mutations in *KRAS* [65], indicating that a single alteration of the activity of a pathway member is sufficient to induce misregulation of that pathway. We suggest that the positions of deleterious somatic mutation can be used to probe pathways affected by somatic mutation. When considering the noncoding genome, we hypothesized that accumulation of noncoding somatic mutation in the transcriptional regulatory regions of genes belonging to a single pathway may indicate pathways with a significant noncoding mutation load in a population of liver cancer patients.

**Fig 5:**
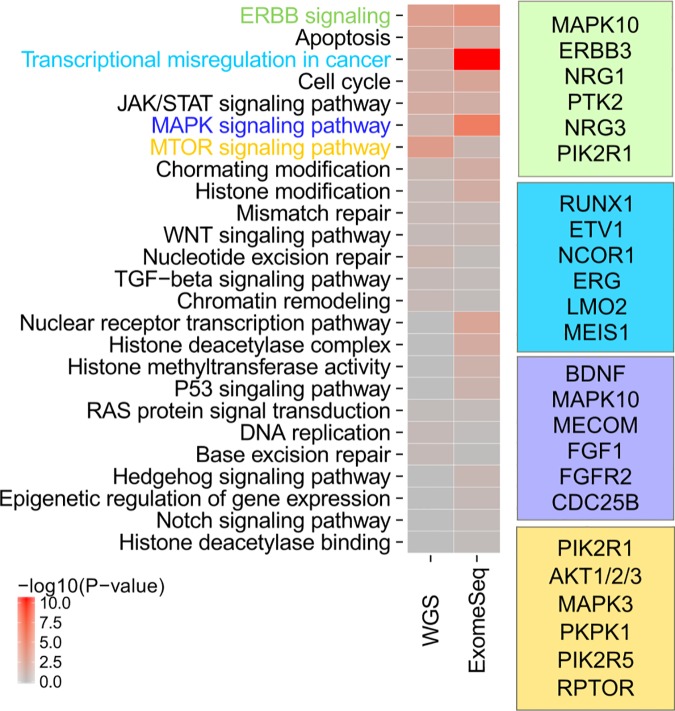
Liver cancer SNV pathway enrichment. Right: Heat map of 25 pathways tested. Color intensity represents the significance of enrichment (–log10(P-value)) for PLC SNVs in promoters that are found in genes for each pathway. WGS = whole genome resequencing-derived PLC SNVs; ExomeSeq = ExomeSeq-derived PLC SNVs. Left: Colored boxes depict a sample of top hits from significantly enriched pathways. Genes listed have the most recurrently hit promoters for the given pathway. Green box = ERBB signaling pathway; blue box = transcriptional misregulation in cancer; purple box = MAPK signaling pathway; gold box = MTOR signaling pathway.

To identify pathways with significant noncoding mutation burden, we first obtained cancer-related pathways as reported in the pan-cancer literature [44] and in liver cancer-specific reports [55,66]. For each pathway, gene lists were collected from publically available databases [67,68],[69]. We then used SNVs assigned to promoters to tabulate the genes hit by somatic regulatory mutation in liver cancer, and identified pathways with a significant noncoding regulatory mutation load in the population of samples tested (**Fig 5**; **Methods**).

In the ExomeSeq data, the most significantly hit pathway was “Transcriptional misregulation in cancer” (KEGG; p-value = 2.67e-11) (**Fig 5**, blue box), a positive result. The next most significant pathway hit was MAPK signaling (p-value = 3.81e-6) (**Fig 5**, purple box; **S4 Fig**). This result was consistent with the finding that five *MAP2K1* regulatory elements were mutated (see above). Additionally, the MAPK pathway is a central regulator of cell growth, so mis-regulation of the MAPK pathway in cancer is not surprising: our data suggest that noncoding mutation may impact MAPK pathway function. Last, the ERBB signaling pathway was significantly mutated (p-value = 1.14e-4) (**Fig 5**, green box; **S5 Fig**).

SNVs from whole genome resequenced PLC samples had fewer pathways significant hit, as the sample size was much smaller. However pathway hits were consistent with the larger, ExomeSeq SNV set. The MTOR signaling pathway was the most significant pathway mutated in this sample set (p = 8.10e-4) (**Fig 5**, gold box). This pathway shares several gene members with the ERBB pathway. Additionally, the ERBB signaling pathway was just under the threshold for significance for the WGS SNV set, after correcting for multiple hypothesis-testing. We anticipate that more samples would replicate the ERBB enrichment result seen for the ExomeSeq SNV set.

## Discussion

Cancer is initiated by sequential somatic mutation or chromosomal structural rearrangements until a cell acquires a selective growth advantage and becomes malignant [1,3,70]. Most characterized somatic mutation is to coding genes, either activating proto-oncogenes or inactivating tumor suppressor genes, and is readily identified by sequence-based methods that detect changes to open reading frames. However, the majority of somatic mutation occurs in noncoding regions [2,29]. Identifying the small fraction of noncoding somatic mutation that has a phenotypic effect remains a challenge, as changes to noncoding regulatory DNA are less straightforward to interpret.

While difficult to detect, mounting evidence suggests that noncoding somatic mutations can act as cancer drivers. Amplification of a locus hosting a proto-oncogene is a common oncogenic mechanism: the *ERBB2* locus is amplified in breast cancer [71] and *EGFR* in glioma multiforme [72,73]. Similarly, amplification of a super-enhancer drives overexpression of oncogenes such as *MYC* and *KLF4* in epithelial cancers [74]. Other structural rearrangements place an enhancer near novel oncogenes, such as *GFI1* and *GFI1b* in subtypes of medulloblastoma [10]. Point mutations can also be detrimental, especially in solid tumors [44]. Point mutations that abrogate cohesion binding sites disrupted chromatin neighborhoods, resulting in mis-regulation of proto-oncogenes by enhancers in neighboring chromatin neighborhoods in T-ALL [12]. In addition, point mutations may create transcription factor binding sites near oncogenes, as has been well-documented at the *TERT* promoter in melanoma, breast cancer, liver cancer, and other diseases [5,7,75–77].

Here we describe an algorithm for filtering noncoding somatic mutation data to arrive at potentially functional SNVs. Our algorithm relies on an empirical measure of hypermutation to remove extremely noisy cancer genomes. Subsequently, epigenomic annotation of variants informed which variants had the potential to modulate transcriptional regulatory states: we found 40.5% of filtered variants occurred in regulatory states in one of the 78 Roadmap Project primary cell and tissue types analyzed. SNVs in liver cancer kept from our filtering method were enriched in regulatory states, especially active promoter states, genic enhancers, and bivalent enhancers and promoters.

The distribution of functional coding mutations per gene in a population tend to be highest in a few, specific genes that vary by disease, while many genes will be infrequently mutated in a population [25,78]. Genes highly recurrently mutated in a disease population are expected to be potent cancer drivers. Alternately, low-frequency recurrently mutated genes are thought to drive cancer by mitigating specific pathways; that is, a single pathway may be mutated in several different ways (by mutation of different genes) across individual patients [1,79]. We hypothesized that noncoding mutation may follow a similar pattern.

We were not surprised that the *TERT* promoter mutation remained the strongest signal in terms of mutation recurrence. However, by continuing to probe the publically PLC samples, we were able to find new, moderately strong signals, including recurrent regulatory mutations for *C1orf61*, *ESRP1*, and *MAP2K1*. By assigning SNV-containing regulatory elements to putative target genes, we showed that the distribution of noncoding mutations in regulatory elements for specific genes qualitatively mirrors that of coding mutations.

A recent effort to analyze resequenced PLC whole genomes identified several recurrent noncoding mutations [80]. Prominently, the *TERT* promoter mutation remained the unambiguous strongest signal among 300 PLC samples. The Fujimoto, *et al*. effort identified several other noncoding mutations, including two lincRNAs and several promoters and UTRs. In the present study, we did not recover these specific alterations; we did recover candidates in similar genomic feature classes. Specifically we suggest that the *MYC* gain-of-binding site in the promoter of *FGF5* represents a top-priority candidate for biological validation.

Pathway level analysis is increasingly an important way to interpret cancer mutations [2,28,44,81]. Genes with a low frequency of coding mutations in a population can still have a functional effect in an individual, and aggregating these low-frequency mutated genes has been used to identify pathways deregulated in hepatocellular carcinoma [56,66,82–84]. To ask if noncoding mutations accumulated across samples at regulatory elements for genes of specific pathways, we examined somatically mutated promoters in the context of cancer-involved biological pathways. We found significant involvement of mutated promoters for MAPK signaling, ERBB signaling, MTOR signaling, and transcriptional mis-regulation in cancer pathways.

The result of our pathway analysis was consistent with literature that reports MTOR and MAPK pathway activation in HCC [83]. Hepatocyte proliferation is spurred in cirrhotic liver cells by activation of the MAPK pathway via transforming growth factor-α or insulin-like growth factor-2 [85]. ExomeSeq studies of HCC samples have also identified the mTOR and MAPK pathways as significantly enriched for coding mutations [56,66,82]. Indeed, both the mTOR and MAPK pathways are well known to be involved in several cancers via coding mutation [2,44].

Recently, Guturu, *et al*., (2016) [86] examined single nucleotide variants (SNVs) that occurred in TFBSs in several individuals. Guturu, 2016 used a different filtering strategy than presented here–the authors identified SNVs at conserved sites, while we used epigenomic annotation as a proxy for likely function. In addition, the personal genomes manuscript examined germline mutation while this study deals specifically with somatic mutation. Guturu examined individual genomes; the data examined here did not provide enough power to recover signal in individual cancer genomes. Nonetheless, we can compare the basic outcomes: that the gene regulation of signaling pathways (this study) or gene ontologies (Guturu, 2016) may be disrupted by SNVs at TFBSs.

Our analysis suggested noncoding mutations might burden the same pathways as coding mutations. In the future, it will be important to explore new, unanticipated pathways that have a high somatic noncoding mutation load. Additionally, including distal enhancers in this analysis can increase the sensitivity and specificity of analyzing regulatory element mutation burden effects at a pathway level; however more robust and reliable distal regulatory element to target promoter assignment is needed for the analysis to have a reasonable signal to noise.

One way noncoding mutation can influence phenotype is by altering transcriptional regulation, for example, by modulating transcription factor binding site affinities. Indeed, gain-of-function events conferred by somatic noncoding mutations have been characterized in estrogen receptor binding sites [60]. We found that 15.6% of whole genome resequenced candidate SNVs created putative gain-of-binding site events while 17.6% resulting in potential loss-of-binding site events, suggesting that a substantial amount of noncoding mutation had a potential effect on transcriptional regulation. Our method recovered transcriptional regulatory alterations at known oncogenes (*FGF5*) and at cell biological pathway genes that are important for tumor cell biology (*ZFAS1* and tumor cell invasion).

As we gain a better understanding of how noncoding somatic mutation alters transcriptional regulation, it will be important to incorporate noncoding somatic mutation information into algorithms that predict network-level mutation burden [87]. Eventually, such information might better inform differential diagnosis and therapeutic recommendations.

## Methods

### Filtering noncoding variants from the Catalog of Somatic Mutations in Cancer

Catalog of Somatic Mutations In Cancer (COSMIC) v77 noncoding variants file <CosmicWGS_NCV.tsv.gz> and the sample metadata file <CosmicWGS_SamplesExport.tsv.gz> were downloaded from the COSMIC database (http://cancer.sanger.ac.uk/cosmic) (13 July 2015) [35]. Noncoding SNVs were then parsed as follows (see also **Fig 1A**):

1. Using custom python code, filter variants for:
  1.1 Variant’s sample ID had primary site metadata for as “liver”
  1.2 Variant not annotated as known variant position in (e.g. in dbSNP or 1000 Genomes; see ref. [35])
  1.3 Variant is a confirmed somatic mutation (e.g. was not observed in matched normal sample)
  1.4 Variant is from a whole genome resequenced sample
2. Then find the distribution of variants per sample. Based on the distribution:
  2.1 Define hypermutated samples as those above the percentile on the ordered set of SNVs / sample where the rate of change between percentiles is the greatest (0.5% resolution). This was the top 2.5% samples.
  2.2 Remove variants from hypermutated samples.

A similar strategy was used for filtering ExomeSeq derived variants by modifying step 1.4 above (**S1B and S1C Fig**).

### ChromHMM-18 enrichment

#### ChromHMM-18 segmentation data

ChromHMM-18 segmentations by the Roadmap Project on hg19 were downloaded from the Roadmap Project data repository (http://egg2.wustl.edu/roadmap/web_portal/chr_state_learning.html#exp_18state; mnemonics bedfiles archive). Each of the 78 (non-ENCODE cell lines) mnemonics bed files were parsed using custom python code for each EID and each ChromHMM-18 state.

#### Calculating observed, expected values of filtered noncoding SNVs in ChromHMM-18 segmentations

For the set of 78 EIDs’ ChromHMM-18 bedfiles, bedops annotateBed function was used to determine the overlap of filtered noncoding SNVs with each ChromHMM-18 state. The total expected SNVs in state *m* in cell type (EID) *n* was calculated using custom R code as follows:

$$E_{m,n}=total\;SNVs\times \frac{total\;ChromHMM\;annotated\;bp\;for\;state\;m\;in\;cell\;type\;n}{total\;ChromHMM\;annotated\;bp\;in\;cell\;type\;n}$$

Then the total observed SNVs in state *m* in cell type *n* was tabulated and compared to expectation to create plot in **Fig 1D**.

### DNaseI shared versus restricted element enrichment analysis

“Delineation of DNaseI-accessible regulatory regions” data were downloaded from the Roadmap Epigenome Project data repository (http://egg2.wustl.edu/roadmap/web_portal/DNase_reg.html#delieation; RData files (hg19 coordinates)). Shared or restricted determination for each DNaseI region was made using the k-centroid clustering algorithm results provided by Roadmap (text files for order of modules at the same URL). Overlap of filtered COSMIC noncoding SNVs with regions in each DNaseI cluster was done in R using GRanges package and custom R code.

### Regulatory element annotation

Cosmic noncoding SNVs kept after filtering were annotated using the UCSC Known Genes track and the GenomicFeatures R package functions and custom R code.

### Assigning noncoding regulatory single nucleotide variants to target gene promoters

#### Single nucleotide variatn-to-regulatory element assignment

First we constructed a merged regulatory epigenome: The merge of all 78 ChromHMM-18 states was compiled (for autosomes only). For each 200bp window in the ChromHMM-18 annotations, a regulatory classification of enhancer, promoter, transcribed, or inert was given based on observations in the 78 ChroMHMM-18 annotations. Priority was as follows: assignment to enhancer states (states 7,8,9,10,11, and 15); promoter state (states 1,2,3,4, and 14); transcribed states (states 5 and 6); inert states (states 12,13,16,17, and 18). Filtered SNVs were assigned to overlapping ChromHMM-18 state regulatory element annotations (enhancer and promoter state regions only) using bedtools. Adjacent regulatory elements were merged and the total number of Cosmic noncoding SNVs / full length element counted using a custom python script. Regulatory elements multiply mutated in the same sample ID were counted as mutated twice, except in the case of adjacent SNVs, which were counted a single nucleotide mutation.

#### Regulatory element-to-target gene assignment

The TxDb.Hsapiens.UCSC.hg19.knownGenes R package was used to construct a transcript database (TxDb) of UCSC known genes. Filtered PLC SNVs were assigned regulatory elements using custom python code. SNV-regulatory elements assignments were read into into R as GRanges object. The start and end of the regulatory elements’ intervals were extended by +/-35kb [46] and overlap with UCSC known promoters was found using the GenomicRanges package mergeByOverlaps function.

### Motif mutation analysis

#### Identifying cancer-related transcription factors and their motifs

Searched PUBMED for transcription factors using the search terms “("transcriptional activator" OR "transcriptional repressor") AND ("transcription factor") AND ("DNA-binding") AND "Homo sapiens"[porgn:__txid9606]”. The resulting list of transcription factor genes was cross-listed the PUBMED-TF set with Cancer Gene Census list [35]. The resulting CGC-TFs list was queried to against JASPAR [88] and TRANSFAC [89] databases to find any motif that is bound by CGC-TFs (106 motifs including heterodimers). For each CGC-TF motif, the position-specific scoring matrix (PSSM) was determined using Biopython tools [90], and threshold PSSM was determined at FPR = 0.001.

#### Motif scanning on wildtype and mutated allele sequences

Sequences were generated for wildtype (hg19 reference) and tumor alleles using custom python code and Biopython modules. For each allele, and for each CGC-TF motif, the log-odds PSSM score that the allele creates the given motif site compared to background nucleotide frequencies was determined using Biopython tools and custom python code. Only PSSM scores above the CGC-TF-specific FPR threshold were kept.

Data were then curated to keep only predicted motif-altering instances with a reasonable effect size: pairs of alleles must have had a PSSM log-odds score > = 2 in at least one allele. The delta value was computed for each pair of wildtype-mutant alleles where delta = mutant allele score–wildtype allele score.

### Pathway analysis

For each set of SNVs (WG resequenced or ExomeSeq derived), SNVs were filtered to retain only those in UCSC Known Gene promoter regions (-2000bp, +500bp from TSS). Gene names of these promoters were retained. Lists of pathway gene members was downloaded from the Molecular Signatures database (MsigDB) [69] (v5.1); pathways selected were from the KEGG [67] or Amigo [68] databases. The retained genes list was intersected with each pathway gene list, and the number of overlapping genes were counted as “hits”.

### Binomial test

A one-sided binomial test was conducted using R for each pathway overlap hits count, where *k* = number of overlapping genes, *n* = number of promoters hit by SNV set, *p* = corrected length of pathway gene list / promoters for UCSC Known Genes (“corrected” as some of the gene symbols in the downloaded pathway gene lists were not present in the UCSC Known Genes track). Bonferroni-correction was used to determine significant p-values.

## Supporting information

S1 Datasets Datasets and URLs used in manuscript. (XLSX)

S1 Fig Data filtering. (a) Top: For COSMIC PLC samples with whole genome resequenced data, each percentile (x-axis) was plotted against the number of SNVs (y-axis). Bottom: Samples ordered from fewest to largest number of SNVs. Red line = cutoff at the greatest rate of change between percentiles. (b) Filtering strategy for COSMIC PLC samples with ExomeSeq data. (c) Same as (a) but for SNVs from PLC samples with ExomeSeq-derived SNVs.
(PDF)

S2 Fig Scale-free distribution of regulatory element mutation. The distribution of SNVs in noncoding regulatory elements versus the number of genes with at least one SNV-containing regulatory element associated with it follows a power law (R^2^ = 0.915).
(PDF)

S3 Fig Delta values from systematic motif detection. (a) Delta values (mutant allele log-odds score–wildtype allele log-odds score) for WGS SNVs before applying threshold criteria. (b) Same as (a) but for ExomeSeq SNVs. (c) ExomeSeq SNVs after applying threshold criteria (at least one score ≥ 2 log-odds over background).
(PDF)

S4 Fig KEGG pathway map for MAPK signaling pathway (hsa04010). Red boxes are genes that have SNV promoter mutations in PLC data. Constructed using Pathway Painter [91]; KEGG map04010 [67] reprinted with permission from Kanehisa Laboratories.
(PDF)

S5 Fig KEGG pathway map for ERBB signaling pathway (hsa04012). Red boxes are genes that have SNV promoter mutations in PLC data. Constructed using Pathway Painter [91]; KEGG map04012 [67] reprinted with permission from Kanehisa Laboratories.
(PDF)

S1 Table Top hit regulatory elements. COSMIC SNVs in the most-hit ChromHMM regulatory elements.
(XLSX)

S2 Table Top hit genes. Numbers of mutated regulatory elements per gene.
(XLSX)

S3 Table Summary statistics. Summary statistics for fold observed/expected SNVs in each ChromHMM-18 state, across 78 cell types.
(XLSX)
