# Supplementary figures and images for: Epigenomic annotation of noncoding mutations identifies mutated pathways in primary liver cancer
Source: PLoS One. 2017 Mar 23;12(3):e0174032. doi: 10.1371/journal.pone.0174032 (PMC5363827; doi:10.1371/journal.pone.0174032)

# Supplementary Figure S1

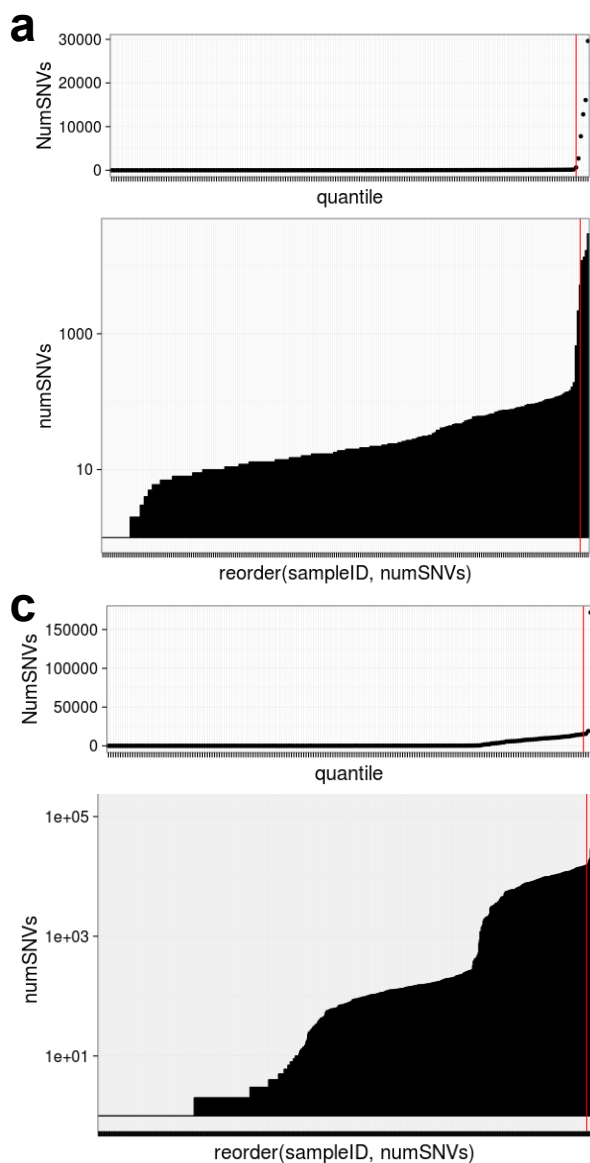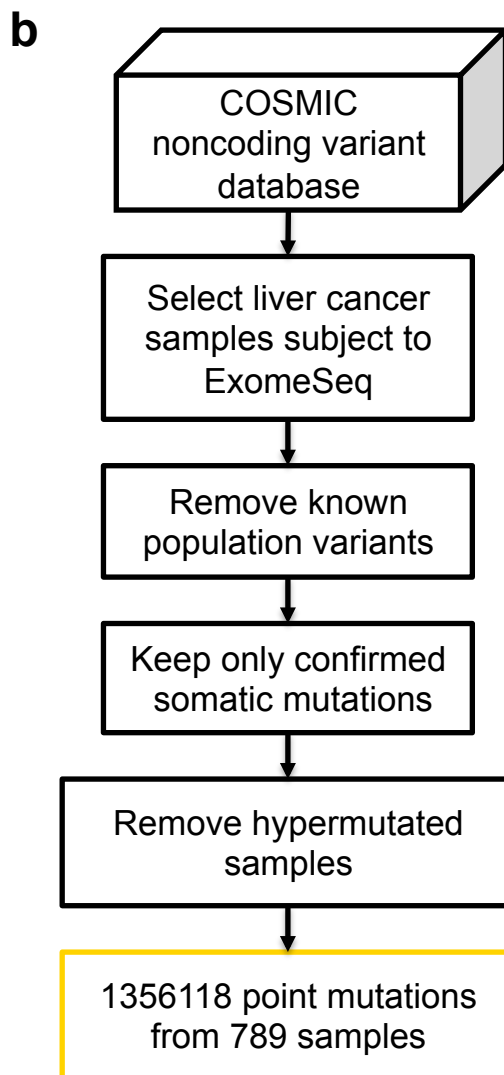

Supplement: S1 Fig — (a) Top: For COSMIC PLC samples with whole genome resequenced data, each percentile (x-axis) was plotted against the number of SNVs (y-axis). Bottom: Samples ordered from fewest to largest number of SNVs. Red line = cutoff at the greatest rate of change between percentiles. (b) Filtering strategy for COSMIC PLC samples with ExomeSeq data. (c) Same as (a) but for SNVs from PLC samples with ExomeSeq-derived SNVs. (PDF) [file pone.0174032.s002.pdf]

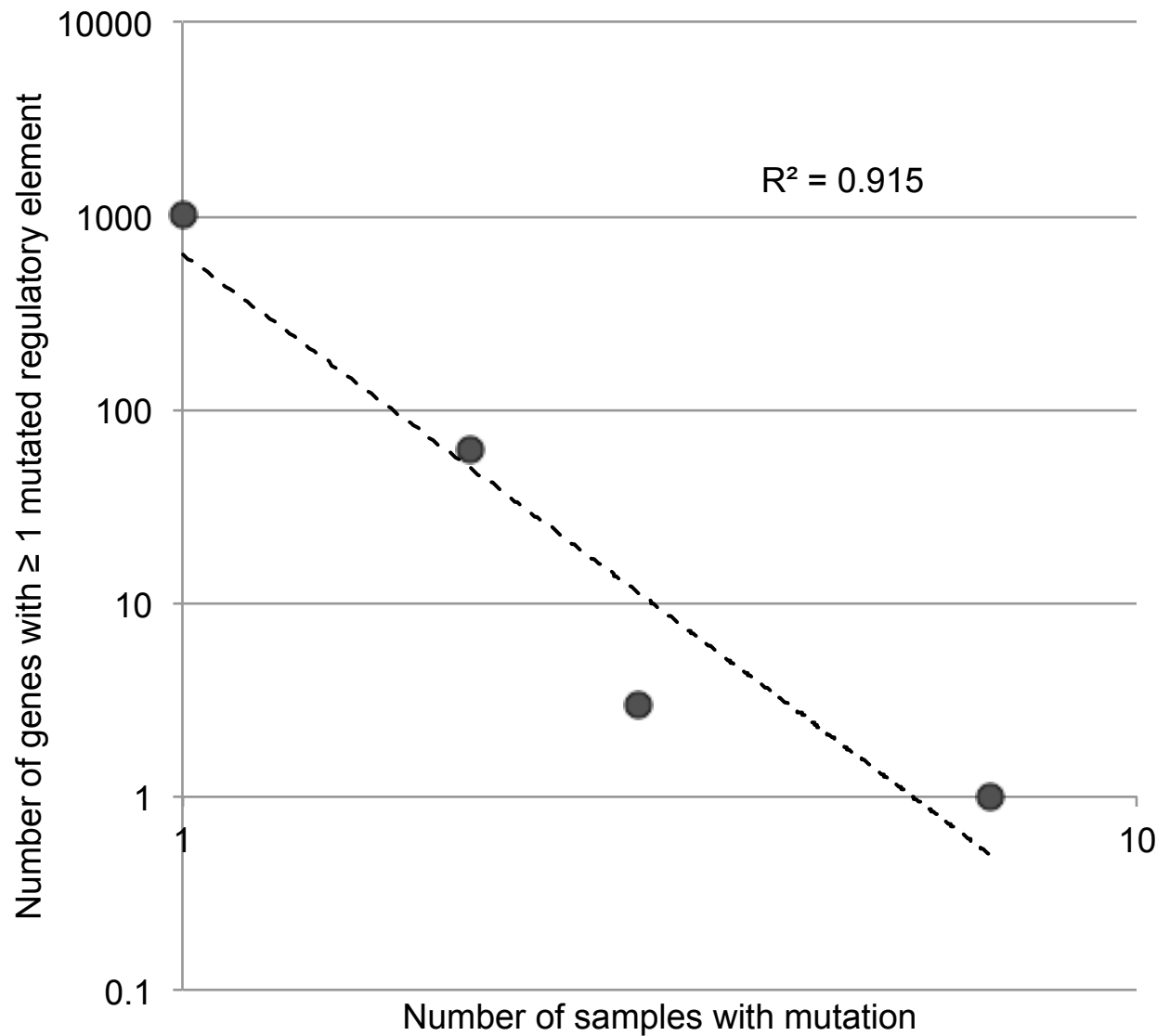

Supplement: S2 Fig — The distribution of SNVs in noncoding regulatory elements versus the number of genes with at least one SNV-containing regulatory element associated with it follows a power law (R2 = 0.915). (PDF) [file pone.0174032.s003.pdf]

Supplemental Figure S2

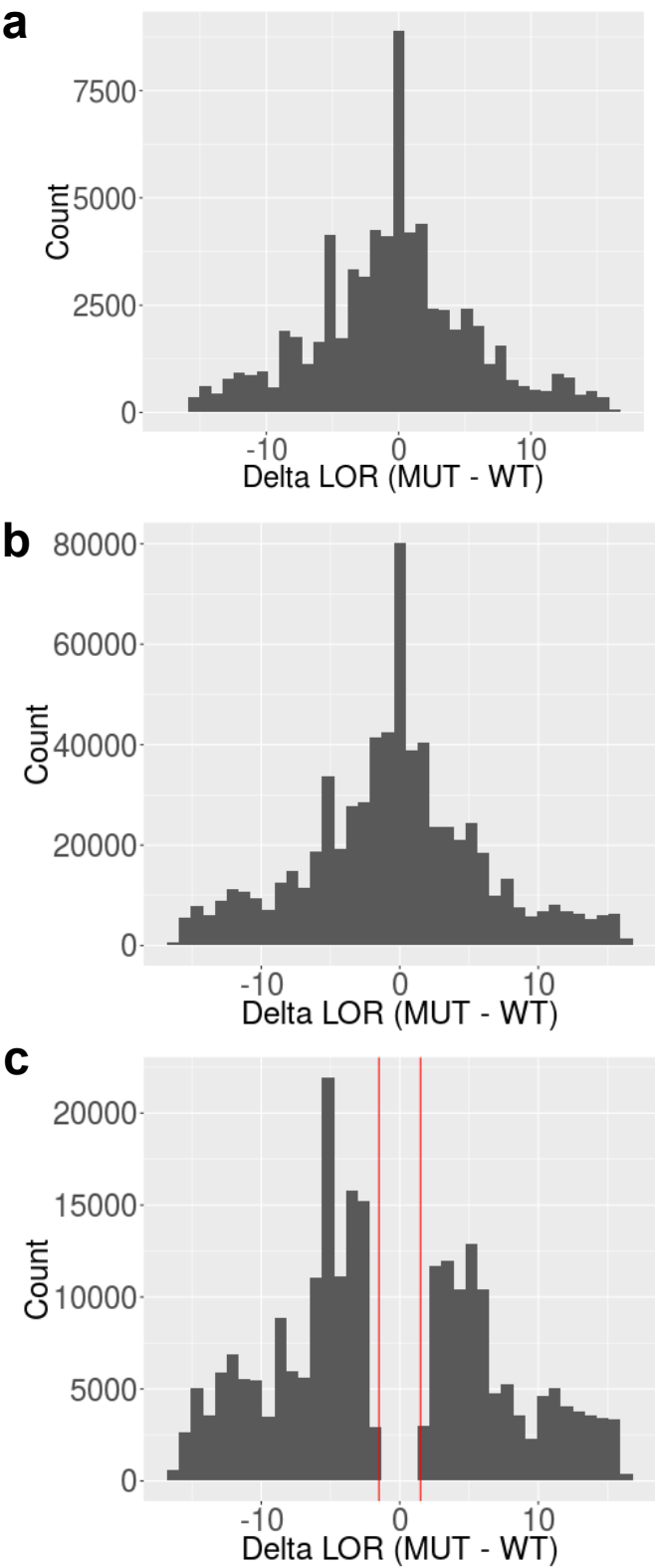

Supplement: S3 Fig — (a) Delta values (mutant allele log-odds score–wildtype allele log-odds score) for WGS SNVs before applying threshold criteria. (b) Same as (a) but for ExomeSeq SNVs. (c) ExomeSeq SNVs after applying threshold criteria (at least one score ≥ 2 log-odds over background). (PDF) [file pone.0174032.s004.pdf]

Supplementary Figure S3

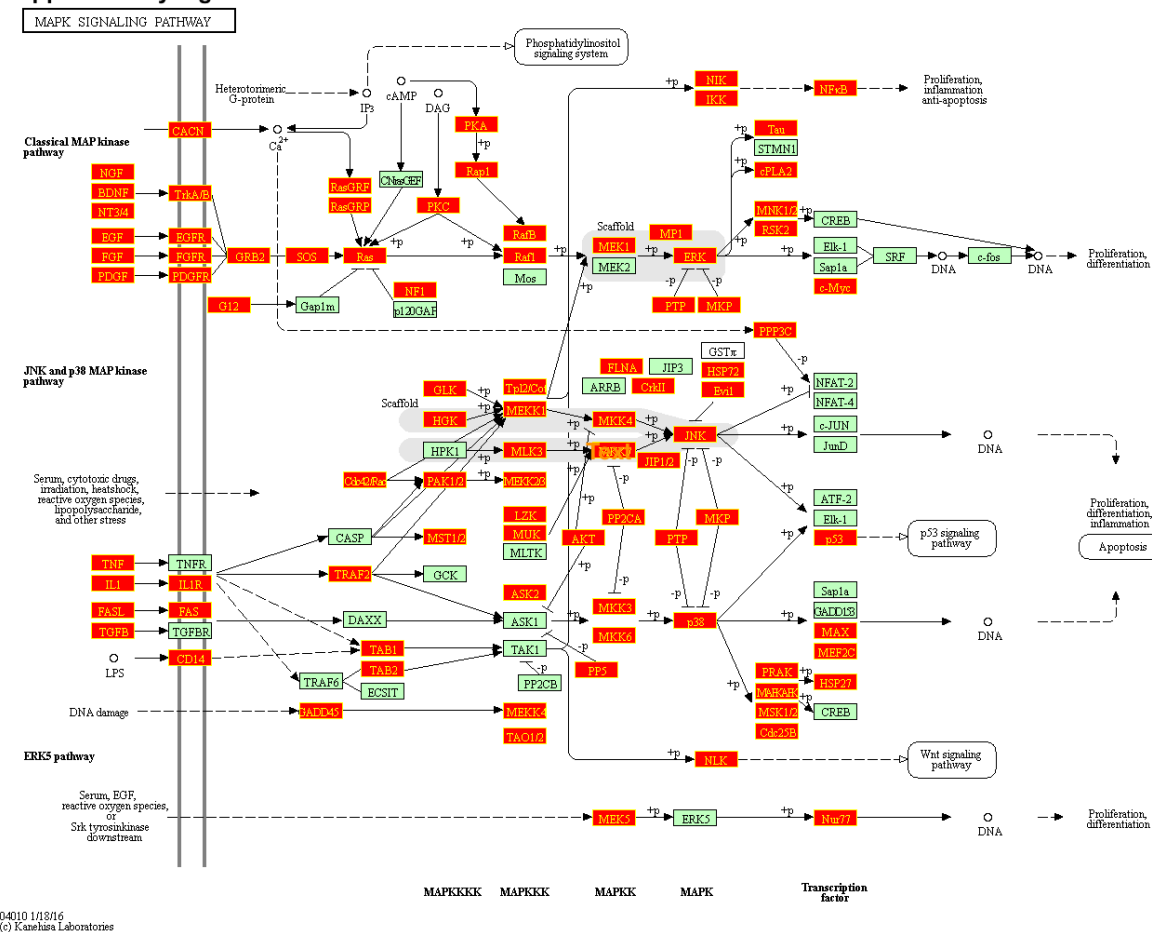

Supplement: S4 Fig — Red boxes are genes that have SNV promoter mutations in PLC data. Constructed using Pathway Painter [91]; KEGG map04010 [67] reprinted with permission from Kanehisa Laboratories. (PDF) [file pone.0174032.s005.pdf]

## Supplementary Figure S4

### ERBB SIGNALING PATHWAY

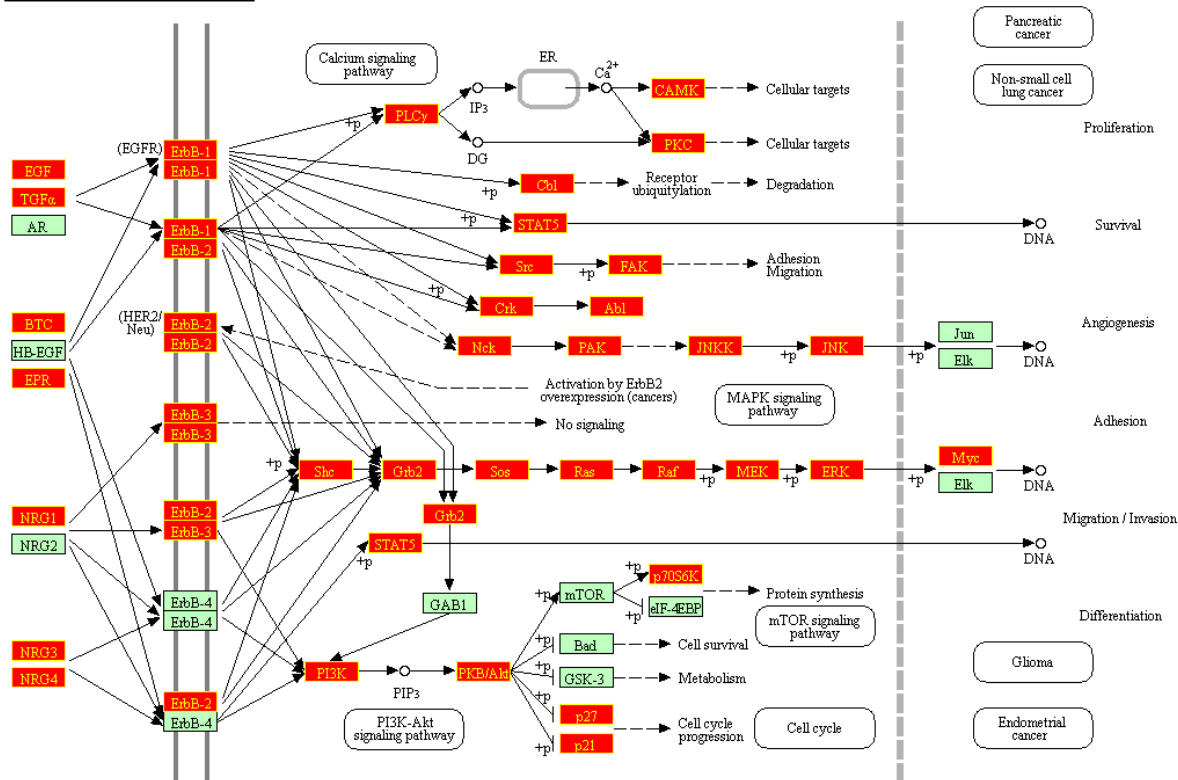

Supplement: S5 Fig — Red boxes are genes that have SNV promoter mutations in PLC data. Constructed using Pathway Painter [91]; KEGG map04012 [67] reprinted with permission from Kanehisa Laboratories. (PDF) [file pone.0174032.s006.pdf]
